# Moderately High Nutrient-Solution Electrical Conductivity Reshapes Stage-Specific Carbon Metabolism Linked to Flavor Formation in Tomato Fruit

**DOI:** 10.3390/ijms27219531

**Published:** 2026-10-25

**Authors:** Zhiyong Shao, Yuyuan Ma, Tonglin Wang, Zhixing Nie, Saisai Guo, Jirong Zheng

**Affiliations:** Vegetable Research Institute, Hangzhou Academy of Agricultural Sciences, Hangzhou 310024, China; shaozy@zju.edu.cn (Z.S.); tlwang@zju.edu.cn (T.W.); niezhixing@126.com (Z.N.); melodygss@163.com (S.G.)

**Keywords:** tomato, nutrient-solution EC, fruit flavor, transcriptome, untargeted metabolomics

## Abstract

Modulating nutrient-solution electrical conductivity (EC) is an important strategy for improving the fruit flavor of tomato (*Solanum lycopersicum* L.) in soilless culture. Previous salinity studies have typically elevated EC through NaCl supplementation, which is physiologically distinct from raising EC *via* basal nutrient concentrations. Moreover, whether the quality-improving effect of moderately high electrical conductivity (HEC) arises from passive osmotic concentration or active metabolic reprogramming remains unclear. Tomato cultivar ‘Hangza 602’ was grown under CK (2.0 mS cm^−1^) and HEC (3.0 mS cm^−1^). Fruits were sampled at mature-green, breaker, pink and red-ripe stages for integrated transcriptomic, enzymatic and metabolomic analyses. HEC increased total soluble solids across ripening stages and promoted sugar and organic acid accumulation at pink and red-ripe stages, with a greater organic-acid increment reducing the sugar–acid ratio. Sugar transporter genes were induced at the breaker stage. At the pink stage, HEC elevated the activities of phosphofructokinase, citrate synthase and NADP–malate dehydrogenase without altering sucrose-phosphate synthase, indicating enhanced catabolic flux rather than sucrose biosynthesis. Sugars and citric acid accumulated persistently, whereas malic acid rose transiently then declined at red-ripe. Multiple downstream pathways were altered in HEC-treated red-ripe fruits. Collectively, HEC initiates stage-specific carbon-metabolic reprogramming combining osmotic and active effects, providing experimental insights for precision EC management in protected tomato production. Future work will further identify key regulatory genes governing this stage-specific metabolic response and validate the combined effects of EC and other environmental factors to develop robust soilless culture strategies for flavor improvement.

## 1. Introduction

Tomato (*Solanum lycopersicum*) is the world’s most valuable vegetable crop, with fruits consumed both fresh and in processed forms [1]. It possesses great industrial value and is widely processed into ketchup, tomato paste, canned tomatoes and tomato juice, supporting a huge global processing industry. Among the numerous traits that determine tomato’s commercial value, fruit flavor quality acts as a decisive trait determining consumer acceptance and market competitiveness [2]. Tomato flavor is a complex trait perceived through the interaction of taste, aroma, and mouthfeel, largely determined by the balance and composition of soluble sugars, organic acids, and volatile organic compounds [3,4,5]. Glucose and fructose represent the major carbon compounds conferring sweet sensation, accompanied by minor contributions from sucrose [2], whereas citric acid (%) and malic acid (%) are the primary metabolites responsible for sour notes [6]. The ratio of total soluble solids (%) to titratable acidity (%) (TSS/TA, sugar–acid ratio) serves as a comprehensive sensory indicator that integrates the relative abundance of sugar and acid pools, and it is widely adopted to systematically assess the harmony and overall palatability of tomato fruit flavor [7,8]. Subtle changes in metabolic accumulation and environmental conditions can dramatically alter sugar–acid homeostasis and ultimately reshape the resulting fruit taste.

In recent years, commercial tomato varieties have commonly exhibited deteriorated flavor performance and reduced sensory quality, which severely restricts market satisfaction and commodity value. Tomato fruit quality formation is comprehensively modulated by multiple cultivation and environmental factors during greenhouse production, making pre-harvest environmental regulation a feasible and efficient approach to optimize fruit flavor characteristics [9,10]. Nutrient solution management, particularly the adjustment of EC, represents one of the most direct and effective agronomic interventions for modulating fruit quality in soilless cultivation systems [11,12]. Reasonable EC management strategies have been proven to effectively reshape the accumulation of soluble sugars and organic acids, thereby improving the flavor quality of horticultural crops. Similar responses have been documented in other greenhouse vegetables. In cucumber, the electrical conductivity of the nutrient solution has been shown to modulate both leaf transcriptome and fruit quality under coir cultivation [13]. Comparative studies across soil and soilless systems further demonstrate that nutrient solution composition significantly influences cucumber fruit quality and physiological characteristics [14]. In chili pepper, salt stress decreased sugar and organic acid contents while increasing capsaicinoid content, with effects dependent on genotype and salinity level [15]. Relevant studies on oriental melon have demonstrated that irrigation amount, nutrient solution EC concentration and irrigation frequency synergistically modulate fruit yield and flavor metabolite accumulation, and the optimized combination of water and EC conditions can simultaneously improve crop yield and fruit flavor quality [16].

For tomato, moderate elevation of nutrient solution EC induces mild salinity stress. This condition does not severely repress plant growth but reshapes primary metabolic pathways within fruit tissues [17,18,19]. Such metabolic shifts promote the build-up of flavor-associated compounds, including soluble sugars, organic acids and functional amino acids; fine-tune the sugar–acid balance; and improve overall taste attributes [20]. Furthermore, higher EC lowers root osmotic potential and reduces root water uptake, mitigating the dilution of dry matter within fruits and further boosting flavor metabolite concentrations [21]. Beyond direct metabolic changes, mild EC-triggered stress remodels whole-plant carbon–nitrogen balance, facilitates photosynthate allocation toward fruits, and enhances both sensory and nutritional properties [22].

However, the beneficial effects of EC are threshold dependent. By contrast, excessively low EC usually leads to vigorous vegetative growth and excessive water accumulation in fruit, as well as dilution of soluble metabolites, and ultimately weakens fruit flavor [23]. Meanwhile, extremely high EC causes severe osmotic stress, restricts plant biomass accumulation, reduces fruit yield, and may even trigger oxidative damage to fruit tissues [24,25]. High EC has also been associated with increased incidence of blossom-end rot and reduced fruit calcium content, further compromising fruit marketability [26]. A series of comparative studies have demonstrated that tomato fruit flavor quality exhibits a threshold-dependent response to EC: it improves under moderately increased EC but declines at excessively high EC levels [27,28].

Consistent with these mechanistic observations, increased nutrient-solution EC has been shown to alter soluble solids, sugar, organic acid and amino acid profiles and improve the sensory acceptance of tomato fruits [29]. Nevertheless, many salinity-oriented studies raise EC through exogenous NaCl addition. Such ionic stress differs substantially from moderate EC elevation achieved by increasing basic nutrient concentrations under practical substrate-culture conditions. Most existing tomato studies focus largely on yield performance and basic quality parameters under single EC treatments [30,31]. Although these works report EC-dependent changes in fruit sugar and acid contents, most conclusions remain empirical. Key carbon-metabolic enzyme activities dictate intracellular carbon flux, yet biochemical data covering successive fruit-ripening stages remain scarce for EC-related fruit-quality research. Many investigations only harvest fully red-ripe fruits and overlook stage-dependent metabolic shifts throughout ripening. It remains poorly clarified whether EC-driven flavor improvement originates merely from passive osmotic concentration effects, or from active reprogramming of central carbon metabolism, including glycolysis and the tricarboxylic acid (TCA) cycle. To address this knowledge gap, multi-dimensional datasets combining dynamic enzyme-activity measurements and untargeted metabolomics are required to dissect the regulatory cascade connecting moderately high-EC treatment and flavor-related metabolite accumulation during tomato fruit ripening.

In the present study, tomato plants were grown under substrate culture with standard-EC control (CK, 2.0 mS cm^−1^) and moderately high-EC (HEC, 3.0 mS cm^−1^) nutrient solutions, and fruits were sampled at four ripening stages. This work aimed to characterize stage-specific carbon-metabolic responses to HEC and uncover how the HEC-modified central carbon metabolism drives primary- and secondary-metabolite changes over fruit ripening. By integrating physiological, enzymatic and metabolomic analyses, this study seeks to elucidate the mechanistic basis of EC-mediated flavor improvement and to provide theoretical references for precise EC management in protected substrate-culture production.

## 2. Results

### 2.1. HEC Treatment Alters Sugar–Acid Balance During Fruit Ripening

**Figure 1:**
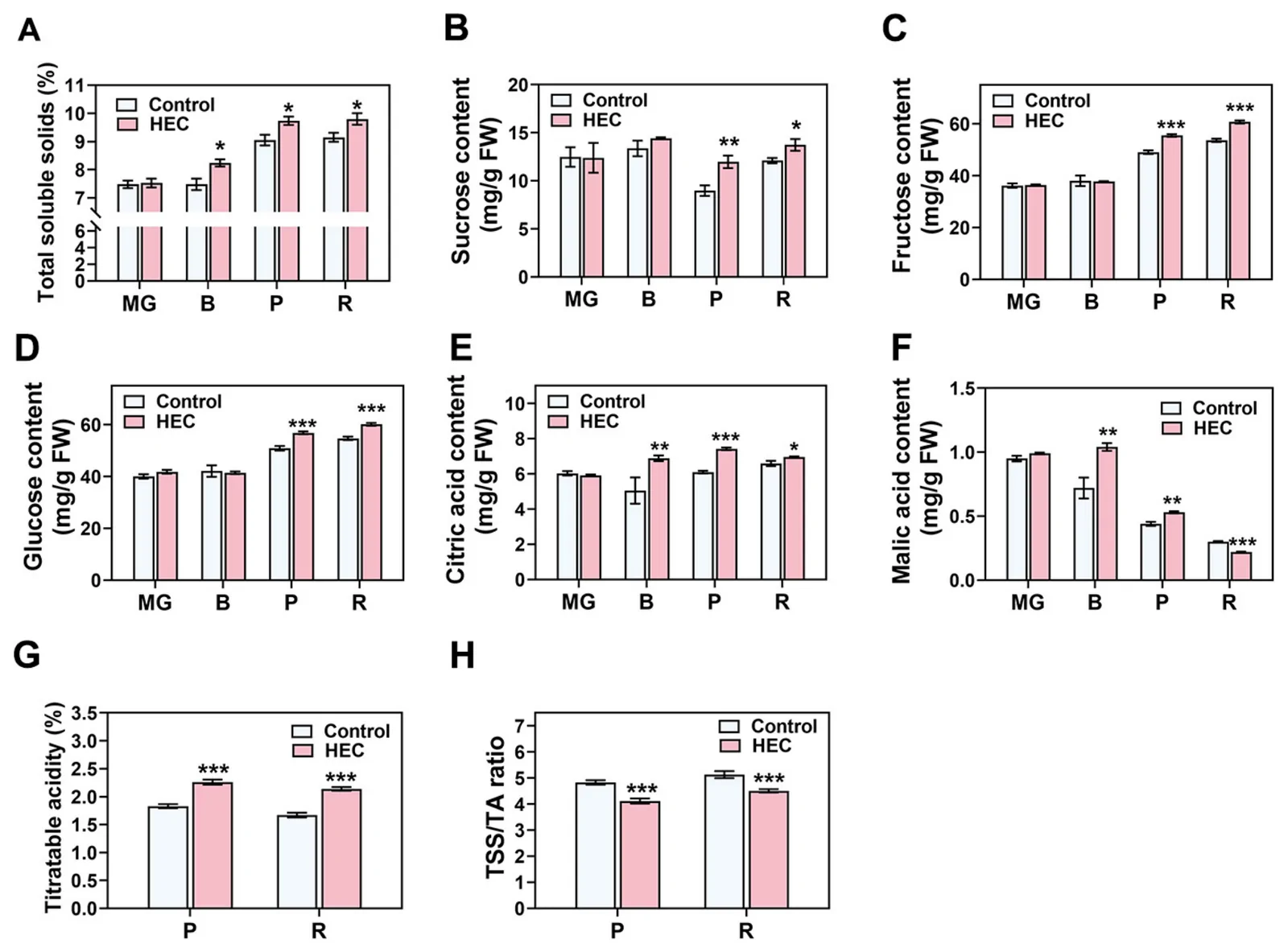
Effects of elevated EC nutrient solution on fruit quality and primary metabolites during tomato ripening: (**A**) TSS; (**B**) Sucrose content; (**C**) Fructose content; (**D**) Glucose content; (**E**) Citric acid content; (**F**) Malic acid content; (**G**) Titratable acidity; and (**H**) TSS/TA ratio. MG, mature green stage; B, breaker stage; P, pink stage; R, red-ripe stage. CK, standard electrical conductivity nutrient solution (EC = 2.0 mS cm^−1^); HEC, moderately high electrical conductivity nutrient solution (EC = 3.0 mS cm^−1^). Data are presented as mean ± standard deviation. Asterisks indicate significant differences between CK and HEC within the same ripening stage (* *p* < 0.05, ** *p* < 0.01, *** *p* < 0.001).

Fruits were harvested at MG, B, P and R stages from plants grown under CK (EC = 2.0 mS cm^−1^) or HEC (EC = 3.0 mS cm^−1^) to evaluate flavor-related primary metabolites (Figure 1A–H). HEC treatment significantly increased TSS from B to R, while no difference was observed at MG. The TSS contents in HEC fruits were 0.42%, 0.55% and 0.60% higher than those in CK at stages B, P and R, respectively. (Figure 1A). The three dominant soluble sugars (sucrose, fructose and glucose) exhibited identical stage-dependent responses to HEC. No significant differences were detected at MG or B for any of these sugars. Sucrose was elevated by HEC at P and R, with increments of 2.99 mg g^−1^ FW at P and 1.64 mg g^−1^ FW at R (Figure 1B). Fructose accumulation was increased by HEC by 6.43 mg g^−1^ FW at P and 7.09 mg g^−1^ FW at R (Figure 1C). Glucose followed a similar pattern, with HEC-treated fruits accumulating 5.95 mg g^−1^ FW (P) and 5.42 mg g^−1^ FW (R) more than CK (Figure 1D).

Citric and malic acids displayed divergent accumulation dynamics in response to elevated EC (Figure 1E,F). Citric acid remained comparable between treatments at MG, but was consistently higher in HEC fruits from B to R, with differences of 1.83, 1.32 and 0.36 mg g^−1^ FW at B, P and R, respectively. Malic acid, in contrast, was significantly elevated by HEC at MG, B and P, yet declined sharply at R, falling from 0.30 (CK) to 0.22 (HEC) mg g^−1^ FW. TA, determined only at P and R, was strongly enhanced by HEC, reaching 2.26% vs. 1.83% at P and 2.14% vs. 1.67% at R (Figure 1G). Although HEC simultaneously increased both soluble sugars and organic acids, the relative increment in organic acids was greater, resulting in a marked reduction of TSS/TA ratio at P (4.83 in CK vs. 4.12 in HEC) and R (5.13 vs. 4.51) (Figure 1H).

### 2.2. Global Transcriptomic Responses in Breaker and Pink Stages

To dissect the molecular regulatory network underlying altered sugar and organic acid metabolism under HEC treatment, RNA-seq was performed on tomato fruit collected at B and P stages from CK and HEC groups, with three biological replicates per treatment. All raw sequencing data passed strict QC (Table S1). Briefly, each library generated 40.91–61.33 million raw reads, and clean reads accounted for over 97% of raw data across all samples. The error rate of each library was maintained at 0.02%, with Q20 ≥ 99.02% and Q30 ≥ 98.06%. The GC content of all samples ranged from 41.95% to 42.45%, indicating stable and high-quality sequencing data suitable for subsequent transcriptional analysis.

**Figure 2:**
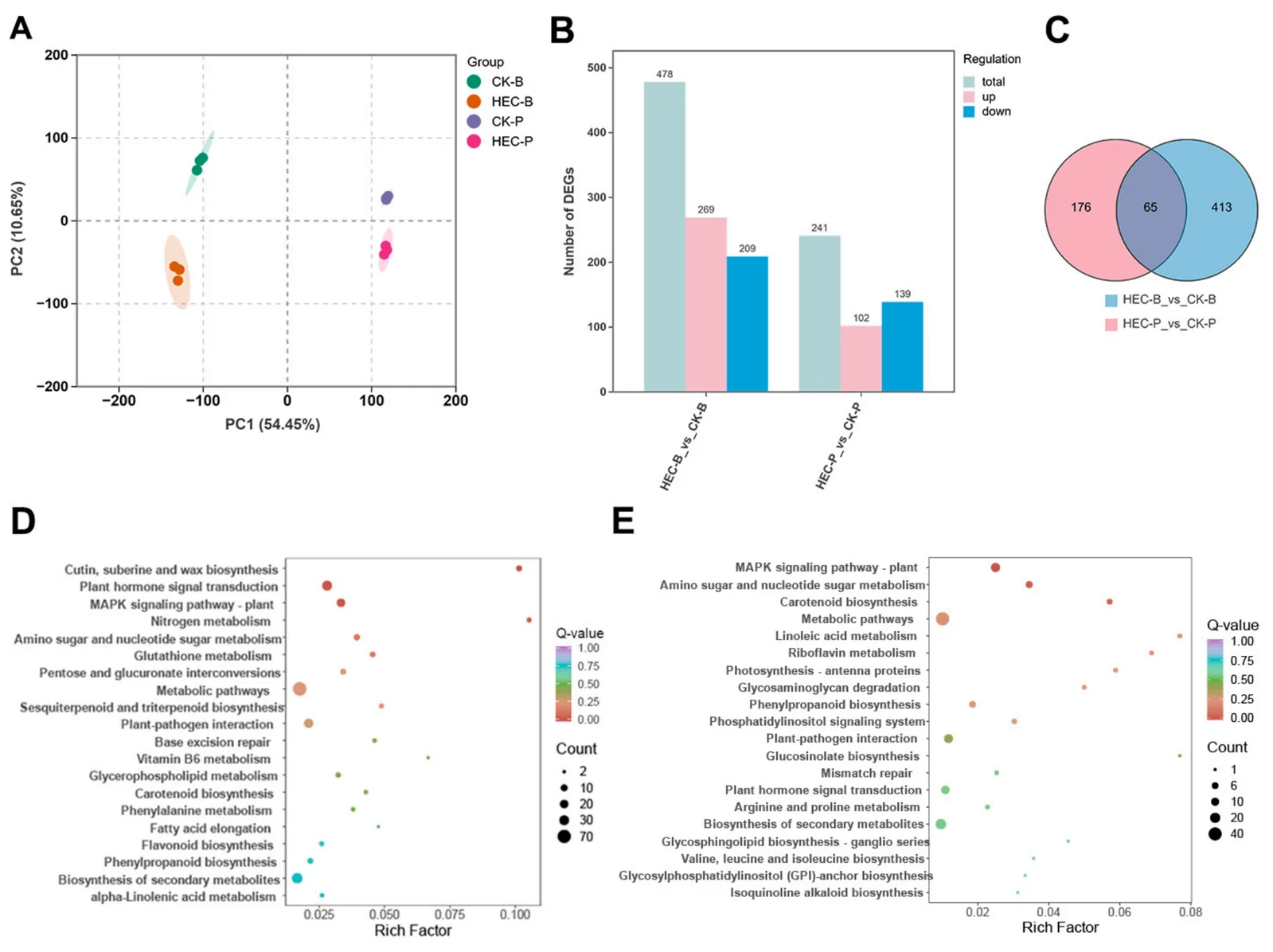
Global transcriptome profiling of tomato fruit at B and P stages under CK and HEC treatments: (**A**) PCA plot showing transcriptional clustering of all RNA-seq samples; (**B**) Bar chart of total, up- and down-regulated DEGs, in two comparison groups; (**C**) Venn diagram displaying overlapping and stage-specific DEGs between B and P stages; (**D**) Bubble plot of top enriched KEGG pathways for DEGs in HEC-B vs. CK-B; and (**E**) Bubble plot of top enriched KEGG pathways for DEGs in HEC-P vs. CK-P. Bubble color gradient represents Q-value, bubble size indicates the number of enriched DEGs.

PCA based on normalized gene expression was conducted to evaluate global transcriptional variation and sample reproducibility (Figure 2A). The samples were visually partitioned into two groups corresponding to B and P ripening stages along PC1 (explaining 54.45% of total variance), demonstrating that fruit developmental progression constituted the dominant driver of transcriptional divergence. Within each ripening cluster, CK and HEC samples formed discrete subclusters, and the spatial separation between CK and HEC was more pronounced at P stage than at B stage, implying stronger transcriptional responses to high EC at P stage. All biological replicates tightly clustered together within each group, confirming excellent repeatability of our RNA-seq datasets. DEGs (differentially expressed genes) were screened with thresholds of |log_2_(FC)| ≥ 1 and adjusted *p* < 0.05 (Table S3). In HEC-B vs. CK-B, a total of 478 DEGs were identified, including 269 up-regulated and 209 down-regulated genes (Figure 2B). For HEC-P vs. CK-P, only 241 DEGs were detected, among which 102 genes were up-regulated and 139 genes were down-regulated. The Venn diagram below further visualizes the overlap and stage-specificity of DEGs between two ripening stages (Figure 2C). A total of 65 DEGs were shared by both B and P stages, representing conserved transcriptional responses to elevated EC throughout fruit color transition. Meanwhile, 413 DEGs were uniquely detected at B stage, and 176 DEGs were specifically responsive to HEC treatment at P stage. The smaller number of P-stage-specific DEGs suggested that most transcriptional responses to high EC at B stage were transient, whereas P-stage-specific DEGs likely participated in persistent regulation of the flavor-related primary metabolism.

Gene Ontology (GO) enrichment analysis was performed to annotate general functional categories of DEGs at B and P stages (Figures S1 and S2). For biological process (BP), cellular process (CC), metabolic process (MF), and response to stimulus were the three most enriched subcategories in both stages. In the cellular component, the cellular anatomical entity and protein-containing complex dominated enriched entries. For molecular function, binding and catalytic activity occupied the largest proportion of enriched DEGs, consistent with dramatic changes in metabolic enzyme activity and primary metabolite pools. Kyoto Encyclopedia of Genes and Genomes (KEGG) pathway enrichment was further applied to characterize core metabolic and signal transduction pathways affected by elevated EC supply (Figure 2D,E). At B stage (Figure 2D), plant hormone signal transduction, mitogen-activated protein kinase (MAPK) signaling pathway—plant, amino sugar and nucleotide sugar metabolism, and nitrogen metabolism were the most significantly enriched pathways, indicating early activation of nutrient sensing and stress signal perception upon high EC treatment before obvious metabolic shifts. At P stage (Figure 2E), amino sugar and nucleotide sugar metabolism, carotenoid biosynthesis, MAPK signaling pathway, and plant hormone signal transduction remained highly enriched. Notably, carotenoid biosynthesis was uniquely and markedly enriched in P-stage DEGs, tightly associated with fruit ripening and color transition, and coordinated with synchronous accumulation of soluble sugars and organic acids in P and R fruit.

### 2.3. Dynamic Expression of Carbon Metabolic Genes and Verification of Key Enzyme Activities

**Figure 3:**
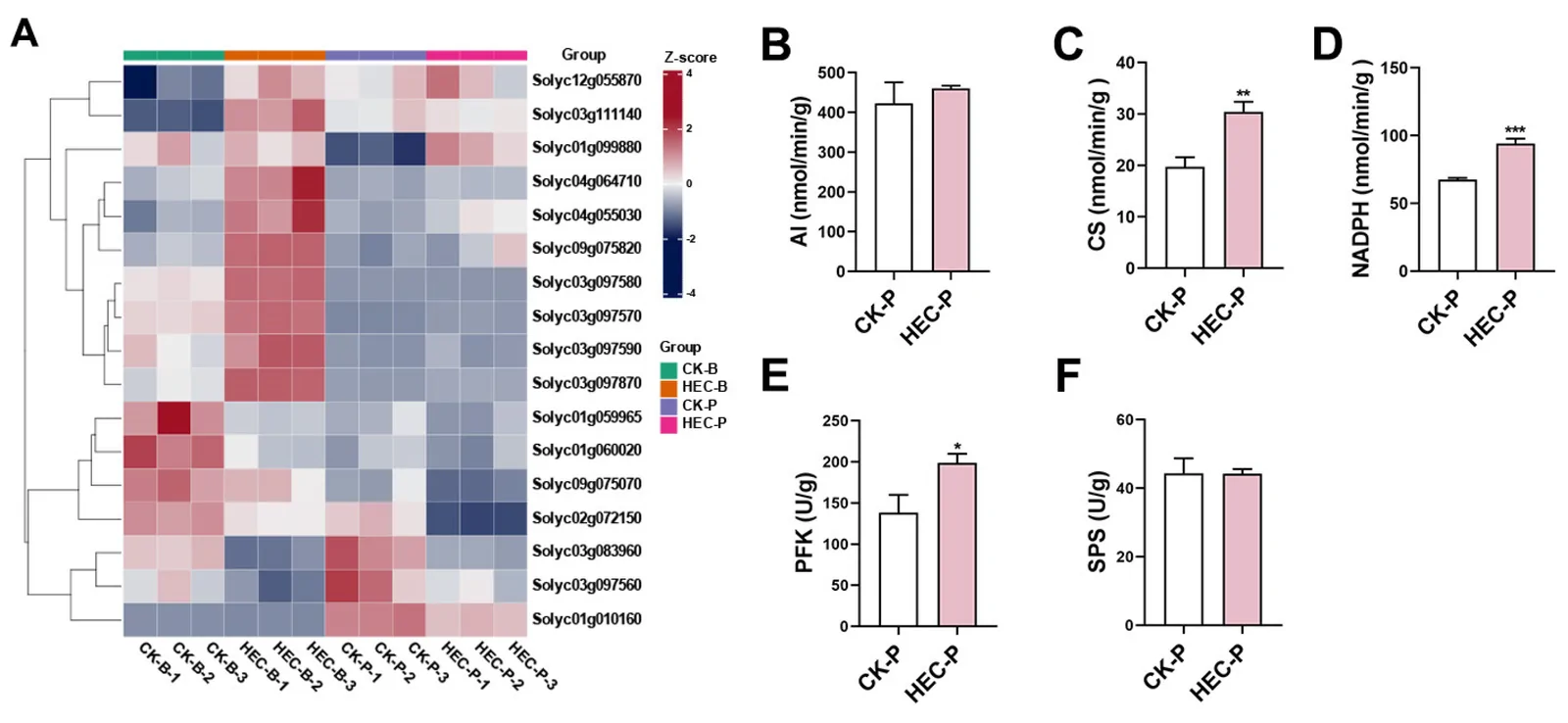
Expression patterns of carbon metabolism-related DEGs and activities of key carbon metabolic enzymes under CK and HEC treatments. (**A**) Heatmap showing the expression profiles of DEGs involved in starch–sucrose metabolism, glycolysis, and TCA cycle. FPKM values were normalized by row-wise Z-score; red indicates high gene expression and blue indicates low gene expression. (**B**–**F**) Activities of five key carbon metabolic enzymes (AI, CS, NADPH, PFK, and SPS) in P-stage tomato fruits. All data are presented as mean ± SD of three biological replicates. Asterisks denote significant differences compared with CK (* *p* < 0.05, ** *p* < 0.01, *** *p* < 0.001).

Consistent with the altered sugar and organic acid accumulation under HEC treatment and the enriched sugar metabolic pathways in transcriptomic profiling, core carbon-metabolism-related DEGs were further analyzed to elucidate the stage-specific carbon metabolic reprogramming induced by HEC. DEGs associated with starch–sucrose metabolism, glycolysis, and TCA cycle were screened, and their dynamic expression patterns were visualized by heatmap, based on row-wise Z-score normalized fragments per kilobase of transcript per million mapped reads (FPKM) values (Figure 3A). Carbon metabolic DEGs exhibited distinct expression characteristics at different ripening stages. Multiple sugar transporter genes, including SWEET and hexose transporter family genes, were significantly induced by HEC at B stage. Genes encoding intracellular carbon metabolic enzymes, including β-glucosidase, TPS, TPP, and SDH, were expressed at low levels at B stage and showed obvious upregulation in HEC-treated fruits at P stage. To validate these transcriptomic observations, four key genes associated with sugar transport and carbon metabolism were subjected to qPCR analysis, and their relative expression levels were quantified across the MG, B and P fruit-ripening stages (Figure S3). Most of these transporter- and carbon-metabolism-related genes were strongly up-regulated by HEC predominantly at B stage, while this inductive effect was attenuated at P stage.

Enzymatic activities of AI, CS, NADPH, PFK, and SPS were determined in P-stage fruits under CK and HEC treatments (Figure 3B–F). Compared with CK-P, HEC-P fruits displayed significantly higher activities of CS, NADPH, and PFK. AI activity increased slightly under HEC treatment, while no significant difference in SPS activity was detected between the two treatments.

### 2.4. Untargeted Metabolomic Analysis of Red-Ripe Fruits

**Figure 4:**
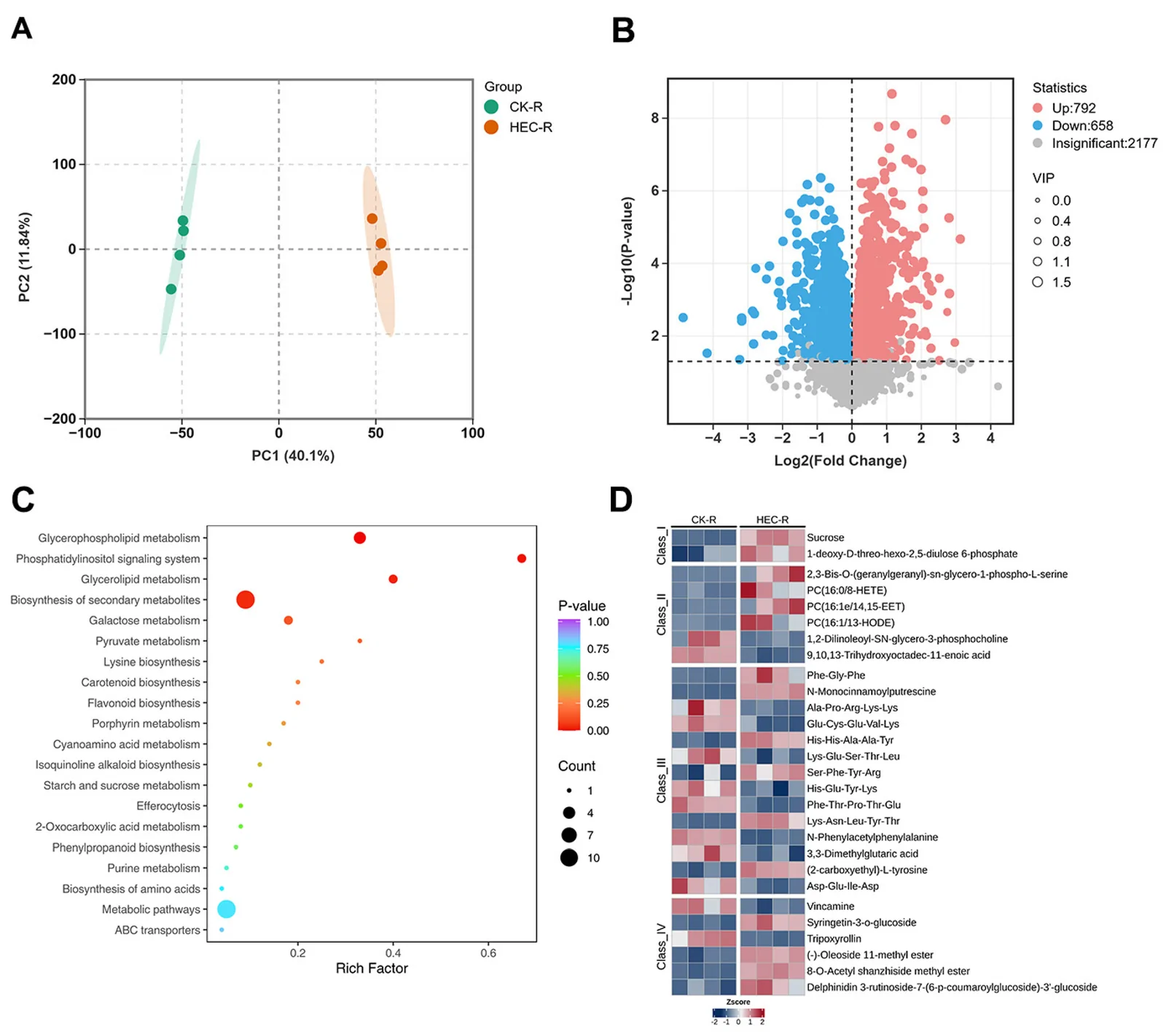
Untargeted metabolomic analysis of red-ripe fruits under high-EC treatment. (**A**) PCA score plot illustrating metabolic differences between CK-R and HEC-R fruit samples. (**B**) Volcano plot of DAMs. Red dots represent metabolites with significantly increased abundance; blue dots represent metabolites with significantly decreased abundance; gray dots indicate metabolites without significant changes. Dot size refers to the VIP value calculated from the OPLS-DA model. (**C**) KEGG enrichment bubble plot based on all DAMs. The *x*-axis shows the rich factor, and the *y*-axis lists enriched KEGG pathway terms. Bubble size corresponds to the count of DAMs assigned to each pathway, and bubble color displays the enrichment *p*-value. (**D**) Z-score-normalized heatmap of selected representative DAMs. Metabolites (rows) were grouped into four functional categories: Class I (sugars and sugar-phosphates), Class II (lipids and phospholipids), Class III (peptides and amino-acid derivatives), and Class IV (secondary metabolites). Columns stand for individual fruit samples from CK-R and HEC-R groups. Red indicates higher relative metabolite abundance whereas blue indicates lower relative abundance. Z-score values range from −2 to +2.

Untargeted metabolomics was used to investigate metabolic changes in red-ripe fruits under high-EC conditions. In the PCA score plot, samples from CK-R and HEC-R were distributed separately along the PC1 axis, with PC1 and PC2 together explaining 52.85% of the total variance. Biological replicates from the same group were located in close proximity, indicating that the high EC treatment altered the metabolite profiles of red-ripe fruits (Figure 4A). In total, 1450 differentially accumulated metabolites (DAMs) were detected, among which 792 were up-regulated and 658 were down-regulated (Figure 4B). KEGG enrichment analysis of all DAMs identified several affected pathways, including glycerophospholipid metabolism, glycerolipid metabolism, biosynthesis of secondary metabolites, galactose metabolism and pyruvate metabolism (Figure 4C). Starch and sucrose metabolism was also enriched, whereas typical soluble sugars (sucrose and glucose) were not found to be significant DAMs in this untargeted dataset. This enrichment signal likely originated from sugar-phosphate and glycoside derivatives. Changes in fruit sugar and organic acid contents have been described in the physiological analyses above. Representative DAMs from four functional groups (sugar-related derivatives, lipids–phospholipids, peptide-amino-acid derivatives, and secondary metabolites) were selected and visualized in a Z-score-normalized heatmap (Figure 4D). Most lipid compounds, peptide derivatives and secondary metabolites accumulated, rising to higher levels in HEC-R fruits. Taken together, these observations show that lipid, peptide-amino-acid and secondary metabolite pathways were widely changed in red-ripe fruits under HEC treatment.

## 3. Discussion

High-EC nutrient solution serves as an effective agronomic approach to improve tomato fruit sensory and nutritional quality, one which modulates sucrose metabolism and promotes sugar accumulation during fruit ripening [32,33]. In the present study, HEC treatment induced distinct stage-specific responses of carbon metabolism in developing tomato fruit. At the early breaker stage, transcriptional shifts of sugar-transporter genes were activated, facilitating carbon resource redistribution inside fruit tissues, whereas obvious biochemical changes occurred at the pink-ripening stage, as represented by elevated enzymatic activities of glycolysis and TCA-cycle-related enzymes. Notably, SPS activity remained unmodified under HEC treatment at the pink-ripening stage, suggesting that enhanced carbon flux was not driven by accelerated sucrose biosynthesis.

Enzyme-activity assays at the pink-ripening stage revealed a clear divergence between catabolic and anabolic carbon metabolism. HEC treatment significantly elevated the activities of PFK, CS and NADP-MDH, all of which contribute to glycolytic and TCA-cycle flux. This pattern indicates that HEC primarily enhances the catabolic arm of carbon metabolism, namely, the breakdown of sugars to generate energy and carbon skeletons, rather than promoting sucrose biosynthesis per se. As a rate-limiting glycolytic enzyme, increased PFK activity directs more carbon flux toward glycolysis, yielding pyruvate and acetyl-CoA for TCA-cycle entry. Although NADP-MDH was induced at the pink-ripening stage and generates oxaloacetate and reducing equivalents, its potential contribution to the red-ripe-stage malate decline warrants further investigation across additional ripening stages to establish causal links.

An important phenomenon revealed in this study is the temporal decoupling between gene transcription and enzyme activity during fruit ripening. Sugar-transporter genes responded to HEC at the transcriptional level at the breaker stage, while significant elevations in PFK and CS enzyme activities only appeared at the pink-ripening stage. Such decoupling between transcript abundance and metabolic activity has also been documented in other fruit-metabolism studies, implying possible post-translational-modification regulation acting on key rate-limiting carbon-metabolic enzymes [34,35]. Transcriptional reprogramming in early ripening fruits establishes the foundation for internal carbon allocation, and subsequent activation of carbon catabolism at the pink-ripening stage supplies sufficient substrates for metabolite synthesis in red-ripe fruits. This two-stage regulatory mode highlights the sequential nature of carbon-metabolic remodeling under HEC.

With this biochemical context in mind, the dynamic changes in soluble sugars and organic acids across ripening stages can be better interpreted. Soluble sugars (sucrose, fructose and glucose) remained unchanged at the breaker stage but increased significantly at pink-ripening and red-ripe stages, demonstrating that HEC-triggered sugar accumulation cannot be simply attributed to passive osmotic concentration. The stage-dependent sugar increase points to active metabolic regulation, consistent with the early-stage up-regulation of SWEET and hexose-transporter genes in HEC-treated fruits. These observations indicate enhanced carbon import into fruit tissues precedes measurable soluble-sugar accumulation. A similar temporal uncoupling between transporter-gene induction and sugar buildup has been reported for salinity-stressed tomato fruits, where vacuolar-sugar-transporter up-regulation preceded hexose accumulation by several days [22].

The disproportionate increase in organic acids relative to sugars also merits attention. Citric acid was consistently elevated by HEC from the breaker to red-ripe stage, while malic acid showed a transient increase at the breaker and pink-ripening stages but declined sharply at the red-ripe stage. This divergent behavior suggests that HEC imposes differential regulatory effects on these two major organic-acid pools. Sustained citric-acid accumulation supports the view of enhanced TCA-cycle flux, whereas late-stage malic-acid reduction may result from its consumption as respiratory substrate or its diversion toward other metabolic pathways during advanced fruit ripening. Comparable malate decreases over tomato ripening have been reported previously and are hypothesized to relate to malic-enzyme and phosphoenolpyruvate-carboxylase activities [36].

Importantly, the metabolic reprogramming observed under HEC cannot be fully explained by osmotic effects alone. Numerous previous studies have shown that moderate salinity improves soluble-solid and soluble-sugar contents in tomato fruits, an outcome frequently attributed to fruit-tissue osmotic dehydration and elevated invertase or sucrose-synthase hydrolysis activity [37]. Distinct from pure NaCl-based salinity stress, HEC nutrient-solution treatment modulates carbon metabolism via both osmotic signals and nutrient-balance effects, prioritizing the activation of glycolysis and TCA-cycle pathways instead of sucrose hydrolysis. Meanwhile, excessive salinity is well-documented as disturbing normal ripening and reducing quality-related metabolite accumulation, which provides a reference for selecting appropriate HEC intensity in practical production [38].

**Figure 5:**
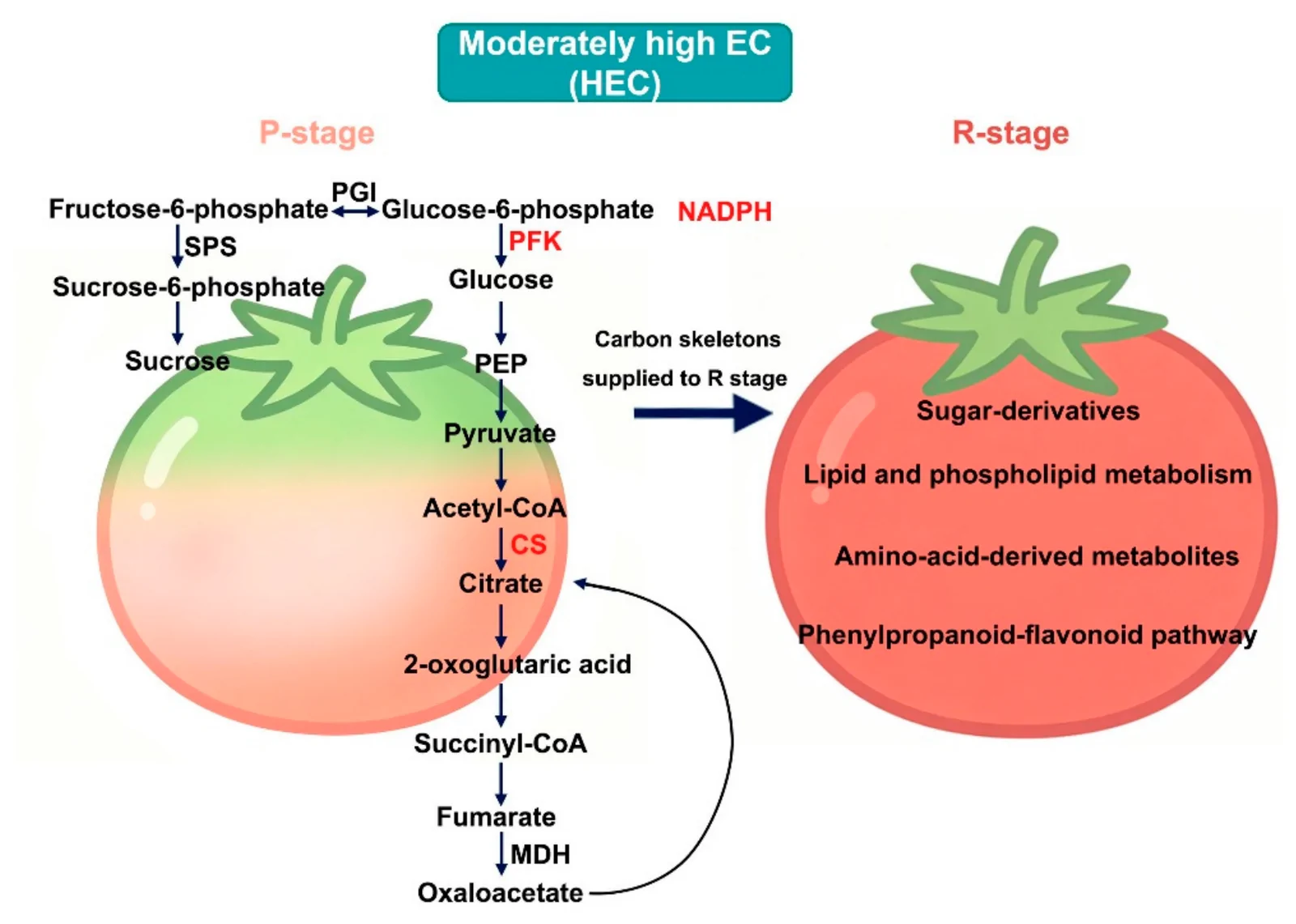
Schematic model illustrating stage-dependent carbon-metabolic reprogramming in HEC-treated tomato fruit. At the pink stage (P-stage), enhanced activities of PFK and CS promote glycolysis and TCA cycle flux to generate carbon skeletons, which are further delivered to the subsequent red-ripe stage (R-stage). These carbon skeletons support multiple downstream metabolic processes, including sugar-derivatives accumulation, lipid–phospholipid metabolism, amino-acid-derived metabolites and the phenylpropanoid-flavonoid pathway. Enzymes marked in red exhibit increased activity under HEC treatment. PFK, phosphofructokinase; CS, citrate synthase; PGI, phosphoglucose isomerase; SPS, sucrose-phosphate synthase; MDH, malate dehydrogenase.

Carbon skeletons generated by enhanced central carbon metabolism serve as essential precursors for both the primary and the secondary metabolism of ripe tomato fruits [39]. Of particular note is the enrichment of phenylpropanoid and flavonoid pathways under HEC. These compounds derive from the shikimate pathway, which draws precursors from glycolytic phosphoenolpyruvate and pentose-phosphate-pathway-derived erythrose-4-phosphate. Higher precursor availability driven by elevated glycolytic flux likely facilitates the accumulation of phenylpropanoid and flavonoid metabolites. Besides improving fruit nutritional quality, these secondary metabolites participate in plant stress defense, implying that HEC may also improve the health-promoting properties of tomato fruits. In agreement with this mechanistic framework, sugar derivatives, lipids, amino-acid derivatives and phenylpropanoid–flavonoids constituted major differentially accumulated metabolites in HEC-treated red-ripe fruits and collectively shape fruit taste, aroma and nutritional value. As summarized in Figure 5, elevated carbon-metabolic flux coincided with robust accumulation of these quality-related metabolites under HEC-CK comparison. Nevertheless, this study mainly characterizes carbon-metabolic alterations triggered by HEC; the upstream hormone-signaling networks mediating these stage-specific metabolic responses remain unelucidated. Further work, such as genetic manipulation of key carbon-metabolic genes, will help establish causal relationships between candidate genes and fruit quality traits.

From the perspective of protected-cultivation application, most previous studies only evaluate fruit-quality phenotypes at full maturity under elevated-EC conditions and overlook dynamic metabolic shifts across successive ripening stages. The stage-specific responses uncovered here demonstrate that fruit developmental status strongly determines metabolic outputs under a given EC regime. Excessively high EC may abolish these beneficial metabolic effects.

These results suggest the possible existence of a mechanism underlying carbon-metabolic remodeling induced by HEC during tomato fruit ripening and provides theoretical support for developing precise nutrient-regulation strategies for protected-substrate-culture tomato production.

## 4. Materials and Methods

### 4.1. Plant Materials and Cultivation Conditions

Tomato (*Solanum lycopersicum* L.) cultivar ‘Hangza 602’ was used as experimental material. In 2025, the experiment was conducted from April to October at the experimental field of Hangzhou Academy of Agricultural Sciences (Hangzhou, China; 30°16′ N, 120°12′ E). Seedlings with consistent growth status were transplanted into substrate culture troughs in a greenhouse. All plants (50 plants per treatment) were irrigated with the standard nutrient solution (CK, EC = 2.0 mS cm^−1^) before the mature green stage. Two nutrient solution EC treatments were initiated at the mature green stage: CK and HEC (elevated EC = 3.0 mS cm^−1^). Each treatment contained three biological replicates with 12 plants per replicate.

Fruits were sampled at four ripening stages: mature green (MG), breaker (B), pink (P) and red-ripe (R) [40,41]. At each stage, uniform fruits without mechanical damage or disease were selected. At the MG stage, fruits are uniformly dark green; at B stage, color break first appears at the blossom-end; at P stage, fruits turn orange-pink; and fully ripe R fruits exhibit homogeneous deep red skin (Figure S4). One biological replicate consisted of 6 fruits harvested from multiple individual plants to avoid bias caused by variation among the fruits of single plants. Three biological replicates were included for each treatment, with 6 fruits per replicate. Fruit was separated, rinsed with distilled water, and divided into two parts: one portion was immediately used for agronomic and flavor index determination; the other portion was frozen in liquid nitrogen and stored at −80 °C for ribonucleic acid sequencing (RNA-seq) library construction and subsequent transcriptional analysis [42]. For RNA-seq sequencing, only fruit at B and P stages was selected, with three biological replicates for each group (CK-B, HEC-B, CK-P, and HEC-P).

### 4.2. Determination of Fruit Flavor-Related Agronomic Traits

The total soluble solids (TSS) (%) of fruit was detected with a digital hand-held refractometer. Titratable acidity (TA) was determined only at P and R stages: 5 g fruit homogenate was diluted with 45 mL distilled water and titrated with 0.1 mol L^−1^ sodium hydroxide (NaOH) to pH 8.1, and TA was calculated based on the citric acid conversion coefficient [43]. The TSS/TA ratio (sugar–acid ratio) was directly determined using a sugar–acid analyzer.

The quantification assay for soluble sugars and organic acids was performed based on the established protocol published by Lisec et al. [44]. Briefly, 0.1 g lyophilized fruit powder was mixed with 1.4 mL methanol, incubated in a thermostatted water bath at 70 °C for 15 min under continuous shaking, then centrifuged at 11,000 *g* for 10 min. The resulting upper aqueous phase was transferred to a new tube and mixed sequentially with 1.5 mL double-distilled water and 750 μL chloroform before thorough vortex mixing, followed by a second centrifugation step at 2200 *g* lasting 10 min. A total volume of 100 μL clarified supernatant was transferred, spiked with ribitol acting as internal standard, and subjected to vacuum drying to remove all solvents. The dried pellet was reconstituted in 60 μL pyridine solution containing 20 g L^−1^ methoxyamine hydrochloride, then incubated at 37 °C for 90 min to complete methoximation. For silylation derivatization, 40 μL mixed reagent of bis-(trimethylsilyl)-trifluoroacetamide (BSTFA) and trimethylchlorosilane (TMCS) (mass ratio = 99:1) was supplemented, and the mixture was maintained at 37 °C for another 30 min. The fully derivatized solution was transferred into sample vials for subsequent gas chromatography (GC) detection. An Agilent 6890 N gas chromatograph system (Palo Alto, CA, USA) fitted with an HP5 capillary column (30 m × 0.25 mm × 0.25 μm, Agilent Technologies) was adopted for metabolite separation. The temperatures of injection port and flame ionization detector were fixed at 250 °C and 280 °C separately. The column heating program was set as follows: initial hold at 100 °C for 60 s; elevated at 2.5 °C per minute to 185 °C; further ramped at 0.35 °C min^−1^ up to 190 °C; heated by 8 °C min^−1^ to 250 °C; finally, increased to 280 °C at a rate of 5 °C min^−1^ and sustained for 3 min. Concentrations of target sugars and organic acids were quantified by external standard calibration curves, and all final data were normalized to fresh weight (FW), presented in units of g kg^−1^ FW.

### 4.3. RNA Extraction, Library Construction and RNA-Seq

Total RNA of frozen B- and P-stage fruit was extracted using Trizol reagent. RNA integrity was verified by 1% agarose gel electrophoresis, and RNA purity and concentration were detected with Nanodrop 2000 (Thermo Fisher Scientific, Wilmington, NC, USA) and a Qubit 3.0 fluorometer. Qualified RNA samples were used to construct complementary deoxyribonucleic acid (cDNA) libraries, following the manufacturer’s standard protocol [45]. The Illumina NovaSeq platform was adopted for paired-end RNA-seq sequencing.

Raw sequencing reads were processed for quality control (QC): reads containing adapters, ambiguous N bases and low-quality reads were filtered to obtain clean reads. The Q20, Q30, guanine–cytosine (GC) content and mapping rate of each sample were calculated (Table S1). Clean reads were mapped to the tomato reference genome using HISAT2 (version 2.2.0) software for subsequent gene expression quantification [46].

### 4.4. Differentially Expressed Gene (DEG) Screening and Functional Enrichment Analysis

Gene expression level was normalized to transcripts per million (TPM) value [47]. DEGs were screened with thresholds: |log_2_(fold change, FC)| ≥ 1 and adjusted *p* < 0.05. Principal component analysis (PCA) was performed based on the normalized TPM data of all samples to evaluate global transcriptional variation and biological repeat consistency. GO enrichment analysis was carried out for DEGs of two stages; DEGs were classified into three main categories: BP, CC and MF. KEGG pathway enrichment was implemented to annotate core metabolic and signal transduction pathways of DEGs; pathways with Q-value < 0.05 were defined as significantly enriched pathways, and bubble plots were drawn to visualize the top enriched KEGG terms. All visualization plots, including PCA scatter plots, DEG statistics bar charts, Venn diagrams, GO classification bar charts and KEGG enrichment bubble plots, were generated using R software (v4.2.1) and its supporting bioinformatics packages.

### 4.5. Enzyme Activity Assays

The activities of citrate synthase (CS, PYHB9), sucrose-phosphate synthase (SPS, PMHB5), phosphofructokinase (PFK, PMHB7), NADPH–malate dehydrogenase (NADPH-MDH, AMHC3), and acid invertase (AI, PYHC2) were determined using commercial detection kits (Nanjing Mofan Biotechnology Co., Ltd., Nanjing, China). Briefly, frozen tomato fruit samples were ground to powder in liquid nitrogen, homogenized with kit-provided extraction buffer, and centrifuged at 8000 *g*, 4 °C for 10 min to obtain crude enzyme supernatants. All photometric measurements were performed on a SpectraMax ABS microplate reader (Molecular Devices, San Jose, CA, USA). Enzyme activities were calculated following the manufacturer’s protocols and expressed on a fresh-weight basis (nmol min^−1^ g^−1^ or μg min^−1^ g^−1^ FW).

### 4.6. Quantitative Real-Time Polymerase Chain Reaction (qPCR) Verification

Complementary deoxyribonucleic acid (cDNA) synthesized from qualified RNA templates was subjected to quantitative real-time polymerase chain reaction (qPCR) verification. Target gene primers designed with Primer 5 and actin as internal reference were mixed with SYBR Green qPCR Master Mix to prepare a 20 μL reaction system, and amplification together with melting curve detection was completed on a Roche LightCycler 480 instrument (Roche Diagnostics, Rotkreuz, Switzerland). Three biological replicates and three technical replicates were arranged for each sample. Each biological replicate consisted of pooled fruit tissues harvested from multiple individual plants; three technical replicates comprised independent qPCR reactions prepared from aliquots of the same cDNA derived from one biological replicate. Relative transcript expression was calculated via the 2^−ΔΔCt^ method [48], and correlation analysis between qPCR data and RNA-seq transcripts per million (TPM) values was implemented in R software (v4.2.1) to confirm sequencing credibility [41]; all primer sequences are summarized in Table S2.

### 4.7. Untargeted Metabolomics Analysis

Frozen red-ripe tomato fruits were vacuum-freeze-dried and ground to powder (MM 400 ball mill, 30 Hz, 1.5 min). Thirty-milligram powder was extracted with 1500 μL −20 °C pre-cooled 70% methanol aqueous solution containing internal standards; samples were vortex-mixed every 30 s for six cycles. After centrifugation (12,000 rpm, 3 min), supernatants were filtered through 0.22 μm membranes for ultra-performance liquid chromatography–tandem mass spectrometry (UPLC-MS/MS) analysis [49].

Metabolites were separated on a Waters HSS T3 column (1.8 μm, 2.1 mm × 100 mm) using a Shimadzu LC-30A UHPLC system. Mobile phases were 0.1% formic acid–water and 0.1% formic acid–acetonitrile; the column temperature was 40 °C, flow rate 0.40 mL min^−1^, and injection volume 4 μL, with gradient elution as documented. Mass spectra were acquired on a SCIEX TripleTOF 6600+ in both ESI positive and negative modes, with mass range 50–1250 Da for MS1 and 25–1250 Da for MS2.

Raw data were converted to mzML format; this was followed by peak picking and alignment and retention-time correction using XCMS (version 3.20.0). Missing values were imputed by KNN or 1/5-minimum substitution, and peak intensities were corrected with SVR. Metabolites were annotated against in-house and public databases together with metDNA. Metabolites with an identification score > 0.5 and QC CV < 0.5 were kept for downstream analysis.

Data were log_2_-transformed and zero-centered for multivariate analysis. PCA was performed using the base R prcomp function. Orthogonal partial least squares-discriminant analysis (OPLS-DA) was conducted via the MetaboAnalystR package with unit-variance (UV) scaling; a 200-permutation test was applied to avoid model overfitting. Hierarchical clustering was visualized by ComplexHeatmap after UV scaling. Differential accumulated metabolites (DAMs) between HEC-R and CK-R were screened by VIP > 1 and |log_2_(fold-change)| ≥ 1. Identified metabolites were annotated and mapped to KEGG Compound and KEGG Pathway databases for metabolic-pathway interpretation.

### 4.8. Statistical Analysis

All statistical calculations and significance assessments were completed in R software (v4.2.1). The Shapiro–Wilk test was used to verify the normality of data distribution before analysis of variance (ANOVA). When the normality assumption was satisfied, one-way ANOVA was adopted to detect intergroup differences of fruit agronomic indicators, primary metabolite concentrations and transcriptional expression abundance, with EC treatment as the fixed factor and biological replicate as the experimental unit. Multiple comparisons of group mean values were implemented via Tukey’s Honestly Significant Difference (HSD) method, with the significance cutoff set at *p* < 0.05. All graphical outputs related to fruit quality quantification and transcriptome bioinformatics analysis were visualized with R (v4.2.1) and its integrated bioinformatic toolkits.

## 5. Conclusions

This study investigated how moderate HEC reshapes stage-specific central carbon metabolism during tomato fruit ripening. Collectively, the results suggest that HEC may trigger a stage-dependent regulatory cascade of carbon metabolism; transcriptional changes in carbon allocation appear at early ripening, followed by elevated carbon catabolic capacity at the pink stage, which could supply carbon skeletons for downstream metabolic events in red-ripe fruit. These findings indicate HEC may initiate carbon-metabolic reprogramming combining osmotic and active effects, offering experimental insights for precision EC management to improve tomato fruit quality in protected substrate culture.

## Data Availability

The original contributions presented in this study are included in the article/Supplementary Material. Further inquiries can be directed to the corresponding authors.

## Supplementary Materials

The following supporting information can be downloaded at: https://www.mdpi.com/article/10.3390/ijms27219531/s1.

## Abbreviations

The following abbreviations are used in this manuscript:

EC	Electrical conductivity
HEC	High electrical conductivity
TSS	Total soluble solids
TA	Titratable acidity
TMCS	Trimethylchlorosilane
FW	Fresh weight
GC	Guanine-cytosine
TPM	Transcripts per million
PCA	Principal component analysis
GO	Gene Ontology
BP	Biological process
CC	Cellular component
MF	Molecular function
KEGG	Kyoto Encyclopedia of Genes and Genomes
qPCR	Quantitative real-time polymerase chain reaction
cDNA	Complementary deoxyribonucleic acid

## References

[1] Shi C., Chen S., Wang J., Chen W., Sun C., Guo Q., Liao S., Wang H., Mu Y., Shu X. (2026). A tomato telomere-to-telomere super-pangenome empowers stress resilience breeding. Nat. Genet..

[2] Zhang J., Lyu H., Chen J., Cao X., Du R., Ma L., Wang N., Zhu Z., Rao J., Wang J. (2024). Releasing a sugar brake generates sweeter tomato without yield penalty. Nature.

[3] Zhao J., Sauvage C., Zhao J., Bitton F., Bauchet G., Liu D., Huang S., Tieman D.M., Klee H.J., Causse M. (2019). Meta-analysis of genome-wide association studies provides insights into genetic control of tomato flavor. Nat. Commun..

[4] Anđelini M., Major N., Išić N., Kovačević T.K., Ban D., Palčić I., Radunić M., Goreta Ban S. (2023). Sugar and Organic Acid Content Is Dependent on Tomato (*Solanum Lycoperiscum* L.) Peel Color. Horticulturae.

[5] Beckles D.M. (2012). Factors affecting the postharvest soluble solids and sugar content of tomato (*Solanum lycopersicum* L.) fruit. Postharvest Biol. Technol..

[6] Jiang Z., Yu Y., Ren X., Zhang S., Sun S., Wang J., Pan C. (2024). SlPPDK modulates sugar and acid metabolism to influence flavor quality during tomato fruit ripening. Biochem. Biophys. Res. Commun..

[7] Fan Y., Liu X., Pan S., Zhao X., Tan M., Xu M., Song Y., Yan X. (2026). Drivers of consumer preference for cherry tomatoes: Insights from sensory, physicochemical and flavor properties. Food Chem. X.

[8] Xiao X.-K., Liu W.-X., Lu X., Chen C.-R., Zhang X.-Y., Pu X.-J., Wang F. (2026). Integrated metabolomics and transcriptomics analyses provide insights into flavor and color regulation during tomato postharvest ripening. Postharvest Biol. Technol..

[9] Fekadu A., Andarege B. (2024). Analysis of the pre-harvest factors that influence on the postharvest quality attributes of Tomatoes (*Lycopersicon esculentum* Mill.): A systematic review. Sci. Hortic..

[10] Qin Y., Gong A., Liu X., Li N., Ji T., Li J., Yang F. (2024). Testing a Simulation Model for the Response of Tomato Fruit Quality Formation to Temperature and Light in Solar Greenhouses. Plants.

[11] Rodríguez F., Pedreschi R., Fuentealba C., de Kartzow A., Olaeta J.A., Alvaro J.E. (2019). The increase in electrical conductivity of nutrient solution enhances compositional and sensory properties of tomato fruit cv. Patrón. Sci. Hortic..

[12] Auerswald H., Schwarz D., Kornelson C., Krumbein A., Brückner B. (1999). Sensory analysis, sugar and acid content of tomato at different EC values of the nutrient solution. Sci. Hortic..

[13] He L., Xu W., Zhou D., Yan J., Jin H., Zhang H., Cui J., Miao C., Zhang Y., Zhou Q. (2024). The Impact of Nutrient Solution Electrical Conductivity on Leaf Transcriptome Contributing to the Fruit Quality of Cucumber Grown in Coir Cultivation. Int. J. Mol. Sci..

[14] Roosta H.R., Azizabadi S., Mirdehghan S.H. (2026). Response of three bell pepper cultivars to different nutrient solutions in NFT hydroponic system. Sci. Rep..

[15] Zamljen T., Medic A., Hudina M., Veberic R., Slatnar A. (2022). Salt Stress Differentially Affects the Primary and Secondary Metabolism of Peppers (*Capsicum annuum* L.) According to the Genotype, Fruit Part, and Salinity Level. Plants.

[16] Sun T., Zhao Y., Zhou D., Gao Z., Hu X. (2023). Effects of amount, electric conductivity, and frequency of a nutrient solution on melon yield and flavor quality. Sci. Hortic..

[17] Saito T., Matsukura C., Kanayama Y., Kochetov A. (2015). Effect of Salt Stress on the Growth and Fruit Quality of Tomato Plants. Abiotic Stress Biology in Horticultural Plants.

[18] Moya C., Oyanedel E., Verdugo G., Flores M.F., Urrestarazu M., Álvaro J.E. (2017). Increased Electrical Conductivity in Nutrient Solution Management Enhances Dietary and Organoleptic Qualities in Soilless Culture Tomato. HortScience.

[19] Li X.-L., Wang C.-R., Li X.-Y., Yao Y.-X., Hao Y.-J. (2013). Modifications of Kyoho grape berry quality under long-term NaCl treatment. Food Chem..

[20] Li Y., Niu W., Cao X., Wang J., Zhang M., Duan X., Zhang Z. (2019). Effect of soil aeration on root morphology and photosynthetic characteristics of potted tomato plants (*Solanum lycopersicum*) at different NaCl salinity levels. BMC Plant Biol..

[21] Keutgen A.J., Pawelzik E. (2008). Quality and nutritional value of strawberry fruit under long term salt stress. Food Chem..

[22] Yin Y.G., Kobayashi Y., Sanuki A., Kondo S., Fukuda N., Ezura H., Sugaya S., Matsukura C. (2010). Salinity induces carbohydrate accumulation and sugar-regulated starch biosynthetic genes in tomato (*Solanum lycopersicum* L. cv. ‘Micro-Tom’) fruits in an ABA- and osmotic stress-independent manner. J. Exp. Bot..

[23] Albu-Yaron A., Feigin A., Rylski I. (1993). The quality of tomato for canning as affected by combined chloride, nitrate and osmotic potential of the nutrient solution. Plant Foods Hum. Nutr..

[24] Rodríguez-Ortega W.M., Martínez V., Nieves M., Simón I., Lidón V., Fernandez-Zapata J.C., Martinez-Nicolas J.J., Cámara-Zapata J.M., García-Sánchez F. (2019). Agricultural and Physiological Responses of Tomato Plants Grown in Different Soilless Culture Systems with Saline Water under Greenhouse Conditions. Sci. Rep..

[25] Ehret D.L., Usher K., Helmer T., Block G., Steinke D., Frey B., Kuang T., Diarra M. (2013). Tomato fruit antioxidants in relation to salinity and greenhouse climate. J. Agric. Food Chem..

[26] Magán J.J., Gallardo M., Thompson R.B., Lorenzo P. (2008). Effects of salinity on fruit yield and quality of tomato grown in soil-less culture in greenhouses in Mediterranean climatic conditions. Agric. Water Manag..

[27] Mizrahi Y. (1982). Effect of salinity on tomato fruit ripening. Plant Physiol..

[28] Zhang P., Senge M., Dai Y. (2017). Effects of Salinity Stress at Different Growth Stages on Tomato Growth, Yield, and Water-Use Efficiency. Commun. Soil Sci. Plant Anal..

[29] Ou X., Liu D., Liu A., Liu H., Chen R., Zhang Y. (2023). Effects of nutrient solution management modes on fruit production and quality of tomatoes grown in extremely root restriction. Sci. Hortic..

[30] Qu F., Zhang Q., Jiang Z., Zhang C., Zhang Z., Hu X. (2022). Optimizing irrigation and fertilization frequency for greenhouse cucumber grown at different air temperatures using a comprehensive evaluation model. Agric. Water Manag..

[31] Zhu K., Zhao Y., Ma Y., Zhang Q., Kang Z., Hu X. (2022). Drip irrigation strategy for tomatoes grown in greenhouse on the basis of fuzzy Borda and K-means analysis method. Agric. Water Manag..

[32] Wu M., Kubota C. (2008). Effects of high electrical conductivity of nutrient solution and its application timing on lycopene, chlorophyll and sugar concentrations of hydroponic tomatoes during ripening. Sci. Hortic..

[33] Jin L., Jin N., Wang S., Huang S., Yang X., Xu Z., Jiang S., Lyu J., Yu J. (2025). Moderate salt stress aids in the enhancement of nutritional and flavor quality in tomato (*Solanum lycopersicum* L.) fruits. Food Chem. X.

[34] Moing A., Berton T., Roch L., Diarrassouba S., Bernillon S., Arrivault S., Deborde C., Maucourt M., Cabasson C., Bénard C. (2023). Multi-omics quantitative data of tomato fruit unveils regulation modes of least variable metabolites. BMC Plant Biol..

[35] Zhang M., Tan F.Q., Fan Y.J., Wang T.T., Song X., Xie K.D., Wu X.M., Zhang F., Deng X.X., Grosser J.W. (2022). Acetylome reprograming participates in the establishment of fruit metabolism during polyploidization in citrus. Plant Physiol..

[36] Osorio S., Vallarino J.G., Szecowka M., Ufaz S., Tzin V., Angelovici R., Galili G., Fernie A.R. (2013). Alteration of the interconversion of pyruvate and malate in the plastid or cytosol of ripening tomato fruit invokes diverse consequences on sugar but similar effects on cellular organic acid, metabolism, and transitory starch accumulation. Plant Physiol..

[37] Meza S.L.R., Egea I., Massaretto I.L., Morales B., Purgatto E., Egea-Fernández J.M., Bolarin M.C., Flores F.B. (2020). Traditional Tomato Varieties Improve Fruit Quality Without Affecting Fruit Yield Under Moderate Salt Stress. Front. Plant Sci..

[38] Hanson B., May D. (2004). Effect of subsurface drip irrigation on processing tomato yield, water table depth, soil salinity, and profitability. Agric. Water Manag..

[39] Colombié S., Nazaret C., Bénard C., Biais B., Mengin V., Solé M., Fouillen L., Dieuaide-Noubhani M., Mazat J., Beauvoit B. (2015). Modelling central metabolic fluxes by constraint-based optimization reveals metabolic reprogramming of developing *Solanum lycopersicum* (tomato) fruit. Plant J..

[40] Pateraki A., Psillakis E. (2025). Vacuum-assisted headspace solid phase microextraction for monitoring ripening-induced changes in tomato volatile profile. J. Chromatogr. A.

[41] Ma Y., Shao Z., Wang T., Nie Z., Guo S., Zheng J. (2026). Metabolomic and transcriptomic analyses reveal grafting-regulated nutritional and flavor metabolites in tomato. BMC Plant Biol..

[42] Martin L.B., Nicolas P., Matas A.J., Shinozaki Y., Catalá C., Rose J.K. (2016). Laser microdissection of tomato fruit cell and tissue types for transcriptome profiling. Nat. Protoc..

[43] Freitas A.d.L.d., Silva J.C.d., de Souza Vandenberghe L.P., Rogez H., Moura F.G. (2024). Bioactive coating containing harzianic acid: Control of Fusarium oxysporum and preservation of tomato quality. Food Control.

[44] Lisec J., Schauer N., Kopka J., Willmitzer L., Fernie A.R. (2006). Gas chromatography mass spectrometry–based metabolite profiling in plants. Nat. Protoc..

[45] Arshad M., Ma Y., Gao W., Zhang S., Shoaib M., Liu X., Fan Y., Li G., Chuai H., Jiang Y. (2025). Polypropylene microplastic exposure modulates multiple metabolic pathways in tobacco leaves, impacting lignin biosynthesis. Ecotoxicol. Environ. Saf..

[46] Kim D., Paggi J.M., Park C., Bennett C., Salzberg S.L. (2019). Graph-based genome alignment and genotyping with HISAT2 and HISAT-genotype. Nat. Biotechnol..

[47] Wagner G.P., Kin K., Lynch V.J. (2012). Measurement of mRNA abundance using RNA-seq data: RPKM measure is inconsistent among samples. Theory Biosci..

[48] Livak K.J., Schmittgen T.D. (2001). Analysis of relative gene expression data using real-time quantitative PCR and the 2(-Delta Delta C(T)) Method. Methods.

[49] Chen W., Gong L., Guo Z., Wang W., Zhang H., Liu X., Yu S., Xiong L., Luo J. (2013). A novel integrated method for large-scale detection, identification, and quantification of widely targeted metabolites: Application in the study of rice metabolomics. Mol. Plant.

