# Supplementary material for: Moderately High Nutrient-Solution Electrical Conductivity Reshapes Stage-Specific Carbon Metabolism Linked to Flavor Formation in Tomato Fruit
Source: Int J Mol Sci. 2026 Oct 25;27(21):9531. doi: 10.3390/ijms27219531 (PMC13763263; doi:10.3390/ijms27219531)
Supplement: Supplementary file 1 [file ijms-27-09531-s001.zip › Supplementary Figure.pdf]

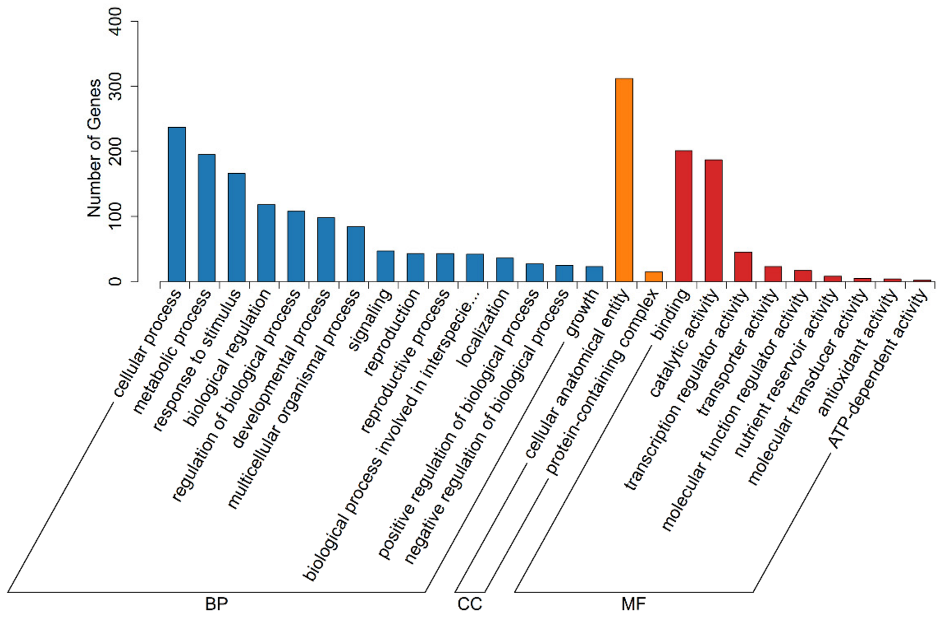

Figure S1. GO classification of DEGs from B-stage fruit (HEC-B vs CK-B). The vertical axis represents the number of enriched DEGs for each secondary GO term, which are grouped into three main categories: biological process (BP), cellular component (CC) and molecular function (MF).

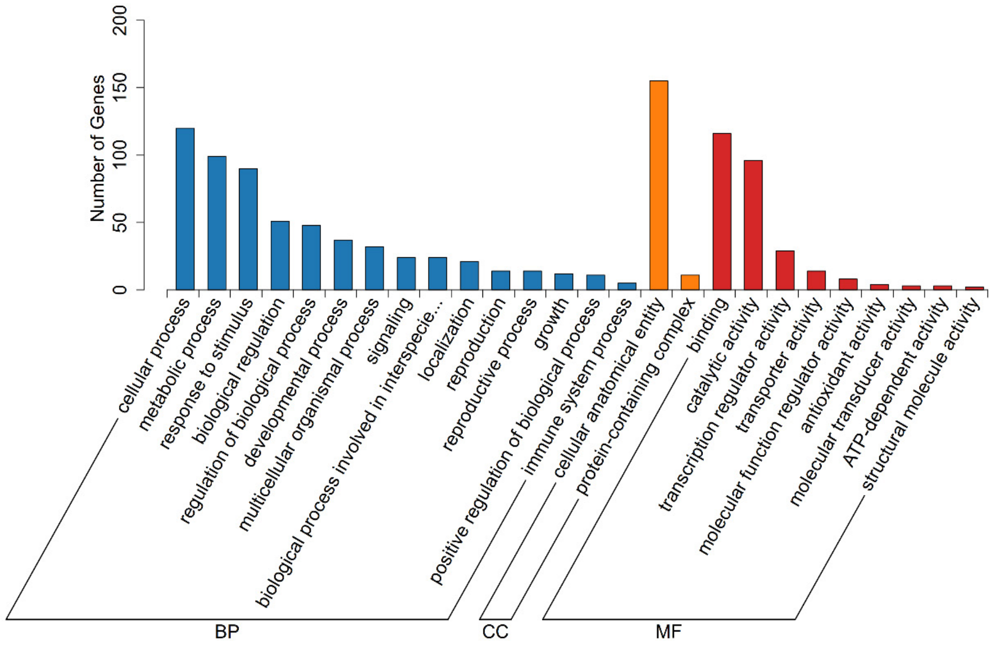

Figure S2. GO classification of DEGs from P-stage fruit (HEC-P vs CK-P). The vertical axis represents the number of enriched DEGs for each secondary GO term, which are grouped into three main categories: BP, CC and MF.

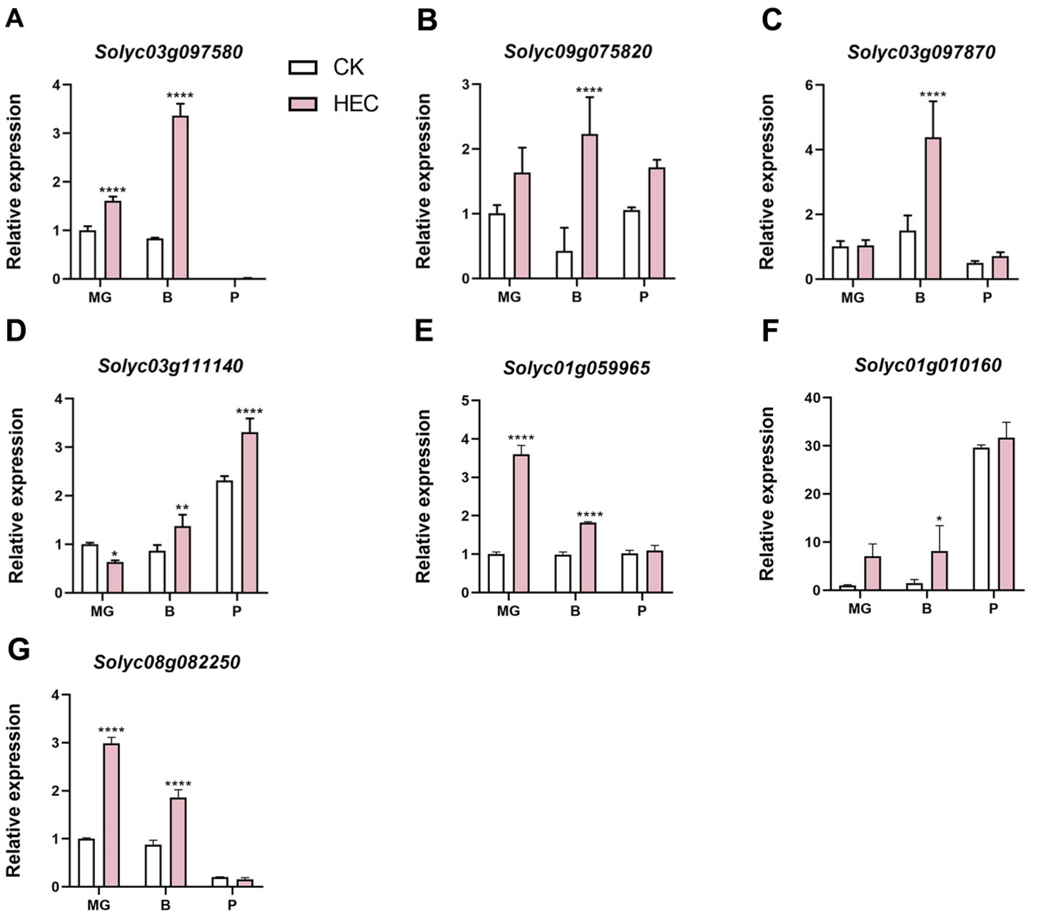

Figure S3. qPCR validation of relative expression levels for selected key carbon-metabolic genes.

Relative expression levels of four representative genes were determined by qPCR across MG, B and P fruit stages under CK and HEC treatments. Data represent mean ± SD of three biological replicates. One-way ANOVA was performed separately within each fruit ripening stage, with EC treatment as the fixed factor. Asterisks denote significant differences between CK and HEC within the same developmental stage: * $P < 0.05$, ** $P < 0.01$, *** $P < 0.001$, **** $P < 0.0001$.

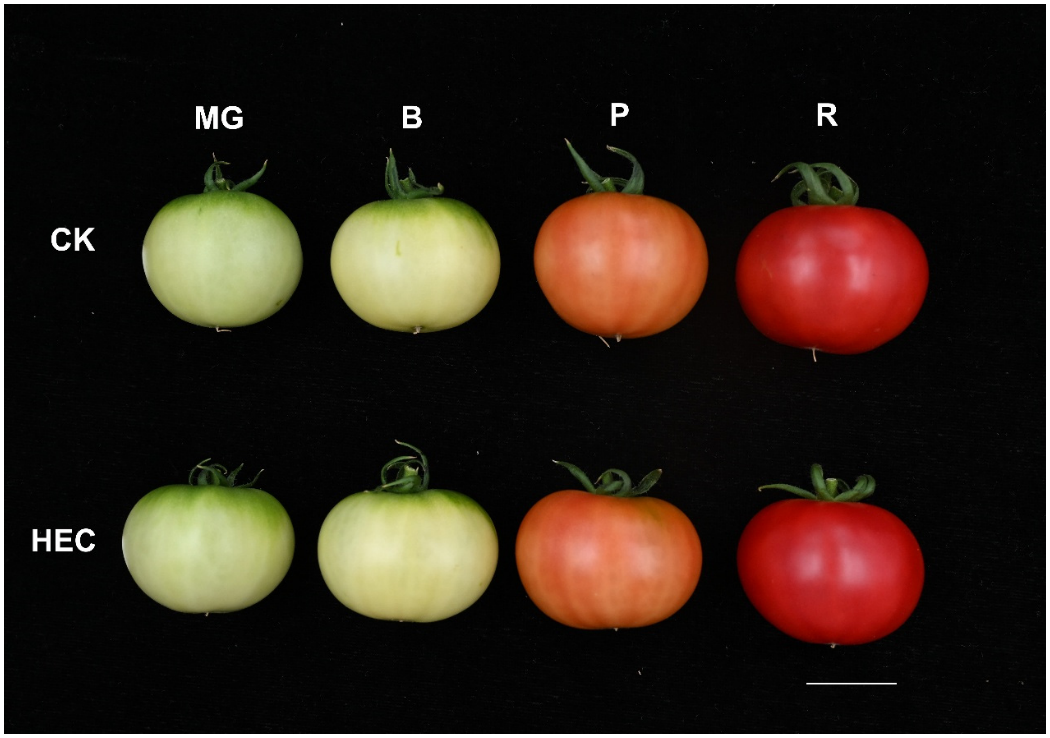

Figure S4. Representative fruit phenotypes of tomato cultivar ‘Hangza 602’ at four ripening stages under CK and HEC treatments.

Mature green (MG), breaker (B), pink (P), and red ripe (R) stages. CK, standard EC nutrient solution (2.0 mS cm^{-1}); HEC, elevated EC nutrient solution (3.0 mS cm^{-1}). The scale bar at the bottom right represents 2.5 cm.
